# mHealth Gratitude Exercise Mindfulness App for Resiliency Among Neonatal Intensive Care Unit Staff: Three-Arm Pretest-Posttest Interventional Study

**DOI:** 10.2196/54561

**Published:** 2024-02-16

**Authors:** Neil E Peterson, Michael Thomas, Stacie Hunsaker, Tevin Stewart, Claire J Collett

**Affiliations:** 1 College of Nursing Brigham Young University Provo, UT United States; 2 Senda LLC Seattle, WA United States

**Keywords:** burnout, compassion fatigue, compassion satisfaction, secondary trauma, trauma, satisfaction, compassion, gratitude, resilience, quality of life, QoL, mindfulness, meditation, exercise, happiness, mHealth, mobile health, app, apps, applications, neonatal intensive care unit, NICU, intensive care unit, ICU, intensive care, nurse, nurses, nursing, health care worker, health care workers, provider, providers, phone app, physical activity, resiliency, mobile phone

## Abstract

**Background:**

Health care is highly complex and can be both emotionally and physically challenging. This can lead health care workers to develop compassion fatigue and burnout (BO), which can negatively affect their well-being and patient care. Higher levels of resilience can potentially prevent compassion fatigue and BO. Strategies that enhance resilience include gratitude, exercise, and mindfulness.

**Objective:**

The purpose of this study was to determine if a 3-week daily resiliency practice, prompted via a gratitude, exercise, and mindfulness smartphone app, impacted the professional quality of life, physical activity, and happiness level of health care workers in a newborn intensive care unit setting.

**Methods:**

In total, 65 participants from a level III newborn intensive care unit at a regional hospital in the western United States completed this study. The Professional Quality of Life Scale, Physical Activity Vital Sign, and Subjective Happiness Score instruments were used to evaluate the effects of the mobile health (mHealth) intervention. Further, 2-tailed dependent paired *t* tests were used to evaluate participant pre- and postintervention instrument scores. Multiple imputation was used to predict scores of participants who practiced an intervention but did not complete the 3 instruments post intervention.

**Results:**

Dependent *t* tests using the original data showed that participants, as a whole, significantly improved in BO (*t*_35_=2.30, *P*=.03), secondary trauma stress (STS; *t*_35_=2.11, *P*=.04), and happiness (*t*_35_=–3.72, *P*<.001) scores. Compassion satisfaction (CS; *t*_35_=–1.94, *P*=.06) and exercise (*t*_35_=–1.71, *P*=.10) were trending toward, but did not reach, significance. Using the original data, only the gratitude intervention group experienced significant improvements (CS, BO, and happiness), likely due to the higher number of participants in this group. Analysis using imputed data showed that participants, as a whole, had significant improvements in all areas: CS (*t*_64_=–4.08, *P*<.001), BO (*t*_64_=3.39, *P*=.001), STS (*t*_64_=4.08, *P*<.001), exercise (*t*_64_=–3.19, *P*=.002), and happiness (*t*_64_=–3.99, *P*<.001). Looking at the intervention groups separately using imputed data, the gratitude group had significant improvements in CS, BO, STS, and happiness; the exercise group had significant improvements in STS and exercise; and the mindfulness group had significant improvements in CS and happiness.

**Conclusions:**

Phone app delivery of resilience-enhancing interventions is a potentially effective intervention model for health care workers. Potential barriers to mHealth strategies are the technical issues that can occur with this type of intervention. Additional longitudinal and experimental studies with larger sample sizes need to be completed to better evaluate this modality.

## Introduction

### Background

Being a nurse is physically and emotionally demanding. Enormous pressure, including overwork, frustration, isolation, and exhaustion from long shifts cause stress, anxiety, and depressive symptoms among health care workers. These psychological stressors not only affect health care workers’ ability to provide competent care but could also have a lasting impact on overall well-being [1]. Compassion fatigue (CF), burnout (BO), and high stress are conditions that can become overwhelming burdens and can cause physical, mental, and emotional difficulties [2,3]. For example, nurses affected by CF may experience dissatisfaction with care, decreased empathy, intolerance to patients, medical mistakes, and leaving the profession [4]. CF and BO may cause a wide range of physical, emotional, and work-related problems that affect both the caregiver and the patient. Medication errors and overall patient safety perceived by nurses have been linked to CF and BO [5]. Nurses who exhibit these negative characteristics may reduce the quality of care, patient safety perceptions, and unit-level safety perceptions [6]. Additionally, elevated levels of BO have been associated with patient dissatisfaction and patient safety concerns [7]. Therefore, it is imperative to address BO and CF as they impact health care workers, patients, and health care systems.

Some nurses are naturally resilient and adapt to stressful work experiences well. Others struggle and strain and may benefit from positive psychology interventions that help build resilience. Resilience is a psychological attribute that is not only intrinsic but can also be learned. It has been recognized as one of the most critical factors in maintaining mental health and psychological well-being among health care workers [8]. Moreover, nurses with high psychological resilience can overcome adversity and adapt to pressures at work [9]. Research supports that individuals can improve their levels of happiness with various emotional management skills [10]. The focus of this study was to empower health care workers to learn skills that may help them cope with working in a high-stress, emotional environment.

### Strategies

Self-care strategies can improve the personal and professional quality of life (ProQOL) of nurses and other health care staff. Further, 3 common interventions to help enhance happiness and resiliency levels include gratitude, exercise, and mindfulness.

First, the simple act of purposefully and consistently expressing gratitude has been found to help people have a greater sense of social cohesion, health, and wellness and is critical to improving well-being [11]. Researchers have known for a long time that expressing gratitude positively affects an individual’s ability to cope and adapt to challenging circumstances [12]. Recent studies reaffirm that gratitude journals can help nurses by improving stress management skills and providing an outlet for self-reflection [13]. Not only does expressing gratitude have a profound effect on the individuals exercising it, but it also influences the people around that individual. Grateful people also improve the environment for others, effectively improving group happiness levels [14].

Second, the positive effects of physical activity on mental health are well-established. Research since the 1990s has established exercise as a simple and effective way to improve many aspects of mental health [15]. Health care givers benefit from exercise as they deal with work stress. Lack of regular exercise may predict the intention to leave work [16]. Recent evidence suggests physical activity can come in various forms, such as daily walks through a hospital garden during work breaks [17] or more structured exercise protocols as part of resilience training [18].

Third, nurses can directly benefit from learning and implementing stress management techniques. Mindfulness through guided meditation is 1 method that has a positive impact [19]. Mindfulness has been shown to help individuals become more self-aware, more open, more accepting of difficult situations, and have more peace. All of these qualities are essential in the prevention of stress and CF in health care workers. Mindfulness is an effective intervention to help improve the ProQOL of health care workers and, because of the link between BO and patient care, may result in more compassionate and patient-centered care [20].

Delivery of resiliency practices is traditionally done during in-person training. However, mHealth delivery of resiliency practices is another option that is effective in many areas including improving mental health and reducing BO in health care professionals [21]. The purpose of this study was to determine if a 3-week daily resiliency practice, prompted via a gratitude, exercise, and mindfulness smartphone app (GEM app), impacted ProQOL, physical activity, and the happiness level of health care workers in a newborn intensive care unit (NICU) setting.

## Methods

### Ethical Considerations

The Intermountain Healthcare’s institutional review board completed and approved the human subjects research ethics review (1051038). Participation was voluntary and participants could withdraw at any time. Compensation of US $25 to a web-based shopping site was given to any participant who completed the prestudy questionnaires. Participants who also completed the poststudy questionnaires were entered into a drawing for a US $100 spa gift card. Participants were assigned code numbers for all data entry purposes. The participant-to-code number list was then destroyed after all study data had been collected. For participant protection, quantitative data are reported in aggregate.

### Sample and Setting

Researchers partnered with a level III, 55-bed NICU at a large local hospital. Institutional review board approval was granted by the hospital system. The NICU had an estimated 160 staff members, counting full-time, part-time, and per diem staff. All adults aged 18 years and older who worked in the NICU with a smartphone compatible with the app were eligible to be in this study. G*Power (version 3.1.9.2; Heinrich-Heine-Universität Düsseldorf) [22] was used to estimate a sufficient sample size for using dependent paired *t* tests, assuming 2-tailed significance, with 80% power and α of .05 with effect size of 0.5. With these assumptions, and treating each group separately, 34 participants were needed for each intervention.

### Data Collection

Recruitment and data collection occurred over 3 months. Before distributing the app, research staff attended several staff meetings to introduce this study to potential participants. Recruitment flyers were posted on the unit with a QR code to download the GEM app. All NICU employees received an email that described this study and included a link to download the iPhone or Android version of the GEM app. Potential participants downloaded the phone app and provided informed consent via the app. After providing consent, study participants completed basic demographic questions and 3 instruments: ProQOL scale, Physical Activity Vital Sign (PAVS), and Subjective Happiness Score (SHS). The GEM app then directed participants to choose 1 of 3 evidence-based resilience interventions: a daily gratitude journal, regular exercise, or mindfulness meditation. The GEM app provided participants daily notification reminders, at a time set by each participant on the app, to engage in their chosen resilience intervention. Participants charted daily completion of their chosen intervention in the GEM app. After 21 days, participants were then prompted to complete the ProQOL, PAVS, and SHS instruments again. The GEM app securely sent all data to a private database maintained by the hospital system.

### Instruments

#### About ProQOL

ProQOL was used to measure both the negative and positive effects of helping others who experience suffering and trauma. ProQOL consists of 30 questions separated into 3 subscales compassion satisfaction (CS), BO, and secondary trauma stress (STS). STS is a measure for CF. The reliability of ProQOL is well-documented and has established its reliability with previous research. It reports a Cronbach score ranging from .71 to .88 [23].

#### About PAVS

PAVS is a validated tool that uses self-reporting to measure exercise levels [24]. PAVS consists of only two questions: (1) on average, how many days per week do you engage in moderate to strenuous physical activity (like a brisk walk)? and (2) on average, for how many minutes do you participate in physical activity at this level? Responses to these questions are then multiplied together to get an estimate of the total number of minutes of physical activity for an average week. Lower scores on the PAVS have been correlated with higher BMI and disease burden [25].

#### About SHS

SHS is a 4-item self-rated measure of general happiness. This instrument has good internal consistency with a Cronbach score ranging from .79 to .94. Test-retest reliability ranged from 0.55 to 0.90, and this instrument was found to have good construct validity [26].

### Data Analysis

Demographics were analyzed using simple descriptive statistics with univariate analysis. Dependent paired *t* tests were done to determine whether the individual interventions impacted participant ProQOL, PAVS, and SHS scores. As this was a pilot study, we did not expect to be fully powered. Thus, missing instrument data were handled using multiple imputations and analyses using original data and imputed data were compared.

## Results

### Demographics

In total, 83 participants downloaded the GEM app and consented to participate in this study. Of those who consented, 65 participants completed their demographics, the 3 instruments, and selected an intervention. After this study, 29 participants did not fully complete the 3 instruments. More participants chose the gratitude intervention (n=32, 49%) than exercise (n=14, 22%) or mindfulness (n=19, 29%). All participants selecting an intervention were women and averaged 1.0 year (SD 8.8 years) of experience on the unit. On average, participants completed their daily intervention on 15.4 out of 21 (73%) days (Table 1).

**Table 1:** Demographics and study characteristics.

Characteristic		Participants, n (%)
**Intervention**		
	Gratitude	32 (49)
	Exercise	14 (22)
	Mindfulness	19 (29)
**Gender**		
	Women	65 (100)
**Unit position**		
	Registered nurse	55 (85)
	Health unit coordinator	5 (8)
	Critical care technician	4 (6)
	Occupational therapist	1 (2)
Age (y), mean (SD, minimum-maximum)		37.9 (10.9, 22-61)
Years working in the unit, mean (SD, minimum-maximum)		1.0 (8.8, 0-39)
Number of days doing intervention, mean (SD, minimum-maximum)		15.4 (7.7, 1-21)

### Attrition and Missing Data

A high number of participants dropped out after downloading the app (21.7%). As this was a pilot study, the decision was made to compare the original data to an imputed data set as an estimate if participants had completed all questionnaires. SPSS (version 25; IBM Corp) was used to perform multiple imputation, using 5 iterations and auto method, on the 29 (44%) participants who practiced an intervention but did not fully complete the 3 instruments on follow-up at the end of this study. Dependent *t* tests were conducted on both the original and the imputed data (Table 2).

**Table 2:** Instrument scores by group.

Group				Original data										Multiple imputation data								
				Participants, n	Pre mean (SD)		Post mean (SD)		2-tailed *t* test^a^ (*df*)		*P* value		Participants, n			Pre mean (SD)		Post mean (SD)		2-tailed *t* test (*df*)		*P* value
**Gratitude group**				18									32									
	**ProQOL^b^**																					
		Comp. satis.^c^			38.8 (4.6)	40.4 (6.2)		–2.40 (17)		.05					38.3 (4.8)		40.1 (5.7)		–3.88 (31)		<.001	
		Burnout			24.3 (5.4)	21.9 (5.6)		2.12 (17)		.05					25.0 (5.2)		23.1 (5.5)		2.99 (31)		.005	
		2° trauma^d^			23.2 (5.6)	21.6 (5.2)		1.10 (17)		.29					24.4 (6.2)		22.1 (5.8)		2.68 (31)		.01	
	**PAVS^e^**																					
		MVPA^f^ min/wk			86.9 (87.8)	117.5 (147.5)		–1.04 (17)		.32					100.5 (95.2)		130.3 (128.4)		–1.81 (31)		.08	
	**SHS^g^**																					
		Happiness			4.8 (1.3)	5.3 (1.3)		–4.04 (17)		<.001					4.8 (1.1)		5.2 (1.4)		–3.13 (31)		.004	
**Exercise group**				7									14									
	**ProQOL**																					
		Comp. satis.			45.0 (4.4)	44.7 (4.8)		0.16 (6)		.88					42.1 (6.7)		42.6 (6.7)		–0.51 (13)		.62	
		Burnout			19.7 (4.6)	19.7 (5.6)		0.00 (6)		1.00					23.2 (6.7)		22.2 (6.2)		1.20 (13)		.25	
		2° trauma			22.4 (6.0)	19.1 (6.1)		2.33 (6)		.06					23.8 (6.3)		21.1 (6.7)		3.52 (13)		.004	
	**PAVS**																					
		MVPA min/wk			74.3 (65.2)	125.7 (51.3)		–1.78 (6)		.13					68.6 (59.6)		108.5 (56.8)		–2.78 (13)		.02	
	**SHS**																					
		Happiness			5.5 (0.9)	5.5 (1.2)		0.00 (6)		1.00					5.1 (1.2)		5.2 (1.2)		–0.97 (13)		.35	
**Mindfulness group**				11									19									
	**ProQOL**																					
		Comp. satis.			36.7 (5.4)	38.0 (5.9)		–1.21 (10)		.26					37.3 (5.2)		38.9 (5.4)		–2.57 (18)		.02	
		Burnout			27.5 (6.2)	25.8 (5.5)		1.27 (10)		.23					26.5 (5.5)		25.4 (5.3)		1.27 (18)		.22	
		2° trauma			28.1 (7.2)	26.2 (7.2)		0.98 (10)		.35					27.4 (6.9)		25.5 (7.5)		1.68 (18)		.11	
	**PAVS**																					
		MVPA min/wk			90.5 (113.9)	10.0 (122.4)		–0.55 (10)		.59					107.6 (99.7)		127.4 (109.2)		–1.92 (18)		.07	
	**SHS**																					
		Happiness			4.2 (1.2)	4.6 (0.8)		–1.75 (10)		.11					4.6 (1.2)		5.0 (1.2)		–2.32 (18)		.03	
**Total, all groups**				36									65									
	**ProQOL**																					
		Comp. satis.			39.4 (5.6)	40.5 (6.2)		–1.94 (35)		.06					38.8 (5.6)		40.3 (6.2)		–4.08 (64)		<.001	
		Burnout			24.4 (6.0)	22.7 (5.9)		2.30 (35)		.03					25.0 (5.7)		23.6 (5.7)		3.39 (64)		.001	
		2° trauma			24.5 (6.5)	22.5 (6.4)		2.11 (35)		.04					25.1 (6.5)		22.9 (6.6)		4.08 (64)		<.001	
	**PAVS**																					
		MVPA min/wk			85.6 (90.6)	113.8 (124.1)		–1.71 (35)		.10					95.7 (90.1)		124.8 (109.8)		–3.19 (64)		.002	
	**SHS**																					
		Happiness			4.7 (1.3)	5.1 (1.2)		–3.72 (35)		<.001					4.8 (1.2)		5.1 (1.3)		–3.99 (64)		<.001	

^a^The *t* test was 2-tailed.

^b^ProQOL: Professional Quality of Life instrument with subscales of compassion satisfaction, burnout, and secondary trauma stress.

^c^Comp. satis.: compassion satisfaction.

^d^2° Trauma: secondary trauma stress.

^e^PAVS: Physical Activity Vital Sign instrument.

^f^MVPA: moderate-to-vigorous physical activity, measured in minutes per week.

^g^SHS: Subjective Happiness Scale.

### Overall Scores

In total, 36 participants had fully complete pre- and postdata in this study. Dependent *t* tests using the original data showed that this group significantly improved their BO (*t*_35_=2.30, *P*=.03), STS (*t*_35_=2.11, *P*=.04), and happiness (*t*_35_=–3.72, *P*<.01) scores. CS (*t*_35_=–1.94, *P*=.06) and exercise (*t*_35_=–1.71, *P*=.10) were trending toward, but did not reach significance. Using the imputed data increased the usable sample size to 65. Analysis of this imputed data showed that all areas had significant improvement (Table 2).

### Gratitude

In total, 32 participants selected the daily gratitude intervention. Of those 32, 18 had complete pre- and postdata. Dependent *t* tests using the original data showed this group significantly improved their CS (*t*_17_=–2.14, *P*=.05), BO (*t*_17_=2.12, *P*=.05), and happiness (*t*_17_=–4.04, *P*<.001) scores. Using the imputed data increased the sample size to 32. CS (*t*_31_=–3.88, *P*<.001), BO (*t*_31_=2.99, *P*=.005), ST (*t*_31_=2.68, *P*=.01), and happiness (*t*_31_=–3.13, *P*=.004) all significantly improved. Exercise was trending toward, but did not reach, significance (*t*_31_=–1.81, *P*=.08; see Table 2).

### Exercise

Further, 14 participants selected the daily exercise intervention. Of those 14, 7 had complete pre- and postdata. Dependent *t* tests using the original data showed this group did not significantly change any of their scores on ProQOL, PAVS, or SHS though ST was almost significant (*t*_6_=2.33, *P*=.06). Using the imputed data increased the sample size to 14. This resulted in significant improvements in ST (*t*_13_=3.52, *P*=.004) and exercise (*t*_13_=–2.78, *P*=.02) but not the other measures (Table 2).

### Mindfulness

Further, 19 participants selected the daily mindfulness intervention. Of those 19, 11 had complete pre- and postdata. Dependent *t* tests using the original data showed that this group did not significantly change any of their scores on ProQOL, PAVS, or SHS. Using the imputed data increased the sample size to 19. This resulted in significant improvements in CS (*t*_18_=–2.57, *P*=.02) and happiness (*t*_18_=–2.32, *P*=.03). Exercise was trending toward, but did not reach, significance (*t*_18_=–1.92, *P*=.07; Table 2).

### Effect Sizes

Cohen *d* was calculated using original and imputed data to estimate the effect size of the GEM app as a whole (combining all intervention group data into one). Using the original data, effect sizes were generally in the low-to-medium range (CS=0.32, BO=0.38, STS=0.35, PAVS=0.29) with one in the medium-to-high range (SHS=0.62). Estimating the effect size using imputed data increased across most measures (CS=0.51, BO=0.42, STS=0.51, PAVS=0.40) except one (SHS=0.49).

## Discussion

### Principal Findings

Overall, this pilot study found that resiliency interventions delivered via mHealth technology are a promising way to improve the well-being of health care workers. When considering only participants who had complete pre- and postdata, only the gratitude intervention group had significant findings (CS, BO, and happiness). When analyzing the imputed data, all intervention groups had significant findings, with the gratitude group experiencing the most significant improvements. It is possible the higher number of participants contributed to the more significant findings of this group. Conversely, the exercise intervention group had the least number of participants. Further, one reason this group may have had fewer numbers is because of the perceived difficulty in comparison to other choices. This would resemble a similar experience by Torquati et al [27] who found nurses were more likely to focus on improving dietary choices rather than physical activity. Despite the lower numbers, those in the exercise group likely made an appropriate choice given that this group had lower average moderate-to-vigorous physical activity at baseline (74.3 min/wk) compared to the gratitude (86.9 min/wk) and mindfulness (90.5 min/wk) groups.

This study strengthens the research that novel delivery of resiliency practices is acceptable to clients and can be used to benefit workers in the high-stress health care environment. For example, Rao and Kemper [28] found that 1-time delivered online training modules for health care workers were well-received and were related to improved gratitude and compassion. Longer-term delivery can also be successful. In another study, a smartphone-delivered mindfulness practice over 3 months provided some benefit to novice nurses over in-person training [29]. Delivery of resiliency practices can also be successfully implemented in other ways. Using gamification and positive competition to recognize the good work of fellow health care coworkers improved gratitude and Press Ganey scores over 18 months [30]. Torquati et al [27] found some success in implementing a phone app combined with a Facebook group to motivate nurses to make positive dietary and physical activity changes over 3 months. While dietary changes were significantly improved, physical activity significantly worsened. Participants reported that trying to improve 2 behaviors at the same time was difficult. In the present study, participants could only choose 1 of the 3 resiliency interventions to follow. As the GEM app improves and research moves beyond the pilot stage, assessing if having multiple intervention offerings is counterproductive will be important to evaluate. Currently, adaptions are already being made to use the GEM app in different contexts, such as for patients who have traumatic brain injury and with millennial caregivers [31].

Researchers considering using mHealth to enhance their studies and improve client outcomes must realize and account for setbacks in preparation and implementation. Development of the GEM app took over double the anticipated amount of time to complete. Further, until recently, most app development had to be done separately for Apple iOS and Android. Now, common programming languages are appearing that make developing for both platforms at the same time possible. This also allows for apps to appear the same on both platforms. Since this was not a possibility when the GEM app was developed, the Apple iOS and Android apps differed in their appearance. The research team attended several staff meetings, posted flyers with QR codes that enabled participants to directly download the app to their phones, and were present in the unit break room at shift change for several days. Despite this preparation, multiple participants struggled with getting started. Having a preassigned research team member providing technical support and establishing an “app support email” was helpful, but this did not resolve all participant issues with the GEM app. Technical issues like this may have contributed to the number of participants who did not fully complete this study.

Imputing data is 1 way to “fill in the gaps” and give a statistical estimation. Although imputing less than 40% is optimal [32], some variables in this study needed up to 44.6% imputation. While this statistical procedure is acceptable for making estimations in a pilot study, going forward we will need to implement strategies to reduce the likelihood of needing this and increase power through adequate participants.

### Conclusion

The purpose of this study was to teach nurses, and other health care staff, evidence-based self-care interventions. Overall, the 3-week daily resiliency practice delivered via the GEM app seemed to positively impact several well-being aspects in NICU health care workers. Practicing these evidence-based interventions can help individuals in similar high-stress work environments experience greater levels of well-being and resilience. While there are multiple positive studies demonstrating the benefits of these self-care interventions, many health care workers do not regularly practice them. This study shows the potential use of mHealth strategies to deliver and develop resiliency habits, such as through the GEM app. Enhancing health care staff resilience can reduce their risk for BO and improve well-being, while also potentially improving the quality of care provided to patients.

## Abbreviations

BO: burnout
CF: compassion fatigue
CS: compassion satisfaction
GEM app: gratitude exercise mindfulness app
NICU: newborn intensive care unit
PAVS: Physical Activity Vital Sign
ProQOL: professional quality of life
SHS: Subjective Happiness Score
STS: Secondary Trauma Stress

## Data Availability

The data sets generated or analyzed during this study are available from the corresponding author upon reasonable request.
